# Prescribing Trends of Promazine in Secondary Care

**DOI:** 10.1192/bjo.2025.10579

**Published:** 2025-06-20

**Authors:** Sunita Edirisooriya, Su Unsal, Nkechi Emelumadu

**Affiliations:** Pennine Care, Bury, United Kingdom

## Abstract

**Aims:** The aim of this audit was to analyse the prescribing trends of promazine in secondary care within a psychiatry department, to evaluate adherence to current guidelines and best practices for promazine prescribing in secondary care and identify potential areas for improvement in prescribing practices to enhance patient care and safety.

Promazine is a phenothiazine-type, first generation (typical) antipsychotic with relatively weak antipsychotic activity making it less effective in treating psychotic disorders but with pronounced sedative effects. It is licensed for short term adjunctive management of psychomotor agitation, and for agitation and restlessness in the elderly. Promazine is highly sedative, has anticholinergic activity, hypotensive effect and can prolong QTc (Corrected QT Interval) at therapeutic doses. Promazine is not endorsed in any recent guidelines for management of agitation e.g. NICE (National Institute for Health and Care Excellence) or BAP (British Association for Psychopharmacology). The BNF (British National Formulary) marks it as a drug considered to be “less suitable for prescribing”.

Despite this, prescribing data suggests it is still being used widely in some areas, particularly in Bury and the North West.

**Methods:** A retrospective review of clinic letters from January 2024 was conducted within the psychiatry outpatient department at Irwell Unit, Fairfield General Hospital. Patients prescribed promazine were identified, and data was collected on diagnosis, indication, duration of use, dose, physical health monitoring, and treatment reviews.

**Results:** Fifty-three patients were prescribed promazine. Of these, 73.6% had no documented indication for its use, and only 3.8% had a documented treatment duration. No patients were on promazine for four weeks or less, the recommended treatment duration. There was no record of review of promazine in a significant proportion (81.1%) of the patients identified. 22.6% had not received any annual physical health checks. Additionally, 41.5% of patients were prescribed promazine alongside another antipsychotic.

**Conclusion:** The audit highlights that promazine is widely being used in the outpatient department at Irwell Unit, Fairfield General Hospital. Its use is not currently in line with local or national guidelines. The findings support the need for improved prescribing protocols, clearer documentation, and regular medication reviews. Action points include increasing awareness through education, ensuring appropriate physical health monitoring, and reviewing all identified patients at their next clinic appointment. A re-audit is planned in six months to assess the impact of these interventions.

